# 6-(2-Chloro­benzyl­amino)purinium tetra­chlorido(dimethyl sulfoxide-κ*O*)(nitrosyl-κ*N*)ruthenate(III) monohydrate

**DOI:** 10.1107/S1600536808006673

**Published:** 2008-03-14

**Authors:** Zdeněk Trávníček, Miroslava Matiková-Maľarová, Kamila Štěpánková

**Affiliations:** a Department of Inorganic Chemistry, Faculty of Science, Palacký University, Křížkovského 10, CZ-771 47 Olomouc, Czech Republic

## Abstract

The asymmetric unit of the title complex salt, (C_12_H_11_ClN_5_)[RuCl_4_(NO)(C_2_H_6_OS)]·H_2_O, contains a 6-(2-chloro­benzyl­amino)purinium cation, a tetra­chlorido(dimethyl sulfoxide)nitro­sylruthenate(III) anion and one solvent water mol­ecule. The Ru^III^ atom is octa­hedrally coordinated by four Cl atoms in the equatorial plane, and by a dimethyl sulfoxide O atom and a nitrosyl N atom in axial positions. The cation is an N3-protonated N7 tautomer. Inter­molecular N–H⋯N hydrogen bonds connect two cations into centrosymmetric dimers, with an N⋯N distance of 2.821 (4) Å. The crystal structure also involves N—H⋯O, N—H⋯Cl and O—H⋯Cl hydrogen bonds.

## Related literature

For related structures of 6-benzyl­amino­purine derivatives, see: Maloň *et al.* (2001 ▶, 2002 ▶); Trávníček *et al.* (2004 ▶, 2005 ▶, 2007 ▶); Trávníček & Matiková-Maľarová (2006 ▶). For the structure of a related Ru complex, see: Serli *et al.* (2002 ▶). For a description of the Cambridge Structural Database, see: Allen (2002 ▶).
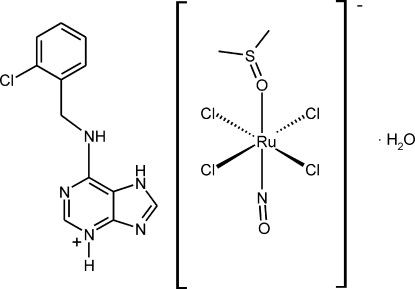


## Experimental

### 

#### Crystal data

(C_12_H_11_ClN_5_)[RuCl_4_(NO)(C_2_H_6_OS)]·H_2_O
*M* *_r_* = 629.73
Orthorhombic, 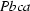

*a* = 15.6229 (5) Å
*b* = 12.8014 (4) Å
*c* = 22.6866 (16) Å
*V* = 4537.2 (4) Å^3^
*Z* = 8
Mo *K*α radiation
μ = 1.40 mm^−1^
*T* = 120 (2) K
0.40 × 0.30 × 0.25 mm

#### Data collection

Oxford Diffraction Xcalibur2 diffractometer with CCD detector
Absorption correction: multi-scan (*CrysAlis RED*; Oxford Diffraction, 2007 ▶) *T*_min_ = 0.604, *T*_max_ = 0.721
36172 measured reflections
3984 independent reflections
3588 reflections with *I* > 2σ(*I*)
*R*_int_ = 0.018

#### Refinement

*R*[*F* ^2^ > 2σ(*F* ^2^)] = 0.031
*wR*(*F* ^2^) = 0.079
*S* = 1.09
3984 reflections
279 parameters
2 restraints
H atoms treated by a mixture of independent and constrained refinement
Δρ_max_ = 1.25 e Å^−3^
Δρ_min_ = −0.58 e Å^−3^

### 

Data collection: *CrysAlis CCD* (Oxford Diffraction, 2007 ▶); cell refinement: *CrysAlis RED* (Oxford Diffraction, 2007 ▶); data reduction: *CrysAlis RED*; program(s) used to solve structure: *SHELXS97* (Sheldrick, 2008 ▶); program(s) used to refine structure: *SHELXL97* (Sheldrick, 2008 ▶); molecular graphics: *DIAMOND* (Brandenburg, 2006 ▶); software used to prepare material for publication: *SHELXL97*.

## Supplementary Material

Crystal structure: contains datablocks I, global. DOI: 10.1107/S1600536808006673/tk2253sup1.cif

Structure factors: contains datablocks I. DOI: 10.1107/S1600536808006673/tk2253Isup2.hkl

Additional supplementary materials:  crystallographic information; 3D view; checkCIF report

## Figures and Tables

**Table 1:** Hydrogen-bond geometry (Å, °)

*D*—H⋯*A*	*D*—H	H⋯*A*	*D*⋯*A*	*D*—H⋯*A*
N3—H3*A*⋯N9^i^	0.88	1.97	2.821 (4)	164
N6—H6*A*⋯O3^ii^	0.88	2.41	3.046 (4)	130
N6—H6*A*⋯Cl2^ii^	0.88	2.66	3.312 (3)	132
N7—H7*A*⋯O3^ii^	0.88	2.45	2.976 (4)	119
N7—H7*A*⋯Cl2^ii^	0.88	2.68	3.290 (3)	127
N7—H7*A*⋯Cl3^ii^	0.88	2.82	3.424 (3)	128
O3—H3*W*⋯Cl3^iii^	0.904 (19)	2.56 (3)	3.386 (3)	152 (4)
O3—H3*V*⋯Cl4^iv^	0.909 (19)	2.61 (3)	3.402 (3)	146 (4)
O3—H3*V*⋯Cl5^iv^	0.909 (19)	2.69 (3)	3.406 (3)	136 (4)

Symmetry codes: (i) 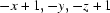; (ii) 
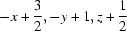
; (iii) 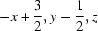; (iv) 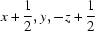.

**Fig. 1.**
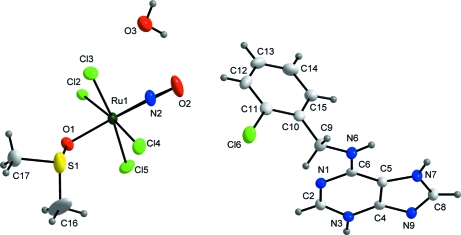
The molecular structure of (I), showing the non-H atoms as 50% probability displacement ellipsoids.

**Fig. 2.**
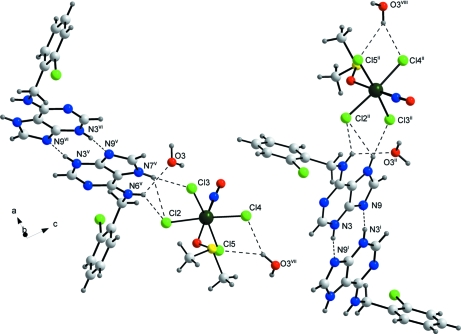
Hydrogen bonding interactions of the type O—H···Cl, N—H···Cl, N–H···O and N—H···N (dashed lines) operating in the crystal structure of (I). Symmetry codes: (i) 1 - x, -y, 1 - z; (ii) 1.5 - x, 1 - y, 1/2 + z; (v) 1.5 - x, 1 - y, -1/2 + z; (vi) 1/2 + x, 1 + y, 0.5 - z; (vii) -1/2 + x, y, 0.5 - z; (viii) 2 - x, 1 - y, 1 - z.

**Fig. 3.**
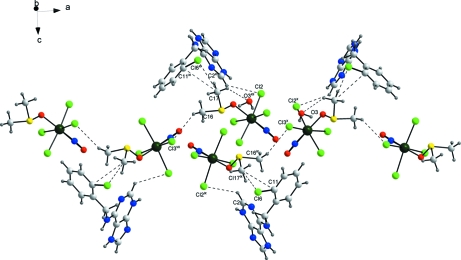
Part of the crystal structure of (I), showing the formation of non-bonding C–H···Cl, C–H···O, C···Cl and C···C (dashed lines) interactions. Symmetry codes: (vii) -1/2 + x, y, 0.5 - z; (ix) 1 - x, -1/2 + y, 0.5 - z; (x) 1.5 - x, -1/2 + y, z; (xi) 1 - x, 1/2 + y, 0.5 - z; (xii) 1.5 - x, 1/2 + y, z.

## supplementary crystallographic information

### Comment

As a part of our systematic study of Ru(III) complexes involving substituted 6-benzylaminopurines, we have prepared the title complex salt, (I), Fig. 1. The structure comprises a 6-(2-chlorobenzylamino)purin-3-ium cation, a [tetrachloro(dimethyl sulfoxide-κO)(nitrosyl-κN)]ruthenate(III) anion and one water molecule of crystallization. The cation exists as the N3-protonated N7 tautomer and contains three different aromatic rings: benzene, pyrimidine (A) and imidazole (B). The A and B rings are nearly co-planar forming a dihedral angle of 1.49 (1)°, while the angle between the benzene ring and purine skeleton (rings A + B) is 85.95 (7)°. The bond lengths and angles in the cation of (I) are similar to those found for 6-(3-chlorobenzylamino)purinium chloride (Maloň et al., 2001), 6-(4-chlorobenzylamino)purinium perchlorate (Maloň et al., 2002), 6-(4-methoxybenzylamino)purinium chloride (Trávníček et al., 2004), 6-(3-methoxybenzylamino)purinium chloride monohydrate (Trávníček et al., 2005), 6-(3-bromobenzylamino)purinium chloride (Trávníček et al., 2006) and 6-(4-hydroxybenzylamino)purinium chloride (Trávníček et al., 2007). Suprisingly, only nine Ru complexes having a RuCl_4_NO coordination geometry have been structurally characterized up to now and deposited in the CSD (Cambridge Structural Database, Version 5.29; Allen, 2002). Moreover, the title complex salt represents only the second X-ray structure determined involving a Ru(NO-κN)Cl_4_(DMSO-κO) moiety.

The geometry about the Ru^III^ atom can be described as a distorted octahedron, as can be seen from the following angles: Cl2-Ru1-Cl4 (174.92 (3)°), Cl3-Ru1-Cl5 (172.39 (3)°), and O1-Ru1-N2 (178.16 (12)°). The N-bonded nitrosyl group occupies a position trans to the O-coordinated dimethyl sulfoxide (DMSO). The Ru–Cl, Ru–N and Ru–O bond lengths of 2.3585 (9)-2.3798 (8), 1.703 (3), and 2.042 (2) Å, respectively, are close to those found for [(DMSO)_2_H][trans-RuCl_4_(NO)(DMSO-κO)] (2.356 (2)-2.373 (2), 1.712 (5), and 2.029 (3) Å, respectively) (Serli et al., 2002).

The O—H···Cl, N—H···Cl, N–H···O and N—H···N hydrogen bonds in (I) contribute to the stabilization of the secondary structure (Table 1, Figs. 2 and 3). Non-bonding interactions of the type C17···C11^xi^ (3.3914 (5) Å), C17···Cl6^xi^ (3.3825 (4) Å), C17—H17A···O3^xii^ (C···O = 3.4419 (5) Å), C16—H16C···Cl3^vii^ (C···Cl = 3.5709 (6) Å) are also present [symmetry codes: xi: 1-x, 0.5+y, 0.5-z; xii: 1.5-x, 0.5+y, z; vii: -0.5+x, y, 0.5-z]. The periodic alternation of anionic and cationic layers in the ac plane can be seen from Fig. 3.

### Experimental

The title complex salt, (I), was prepared by mixing of an ethanolic suspension (2 ml) of 6-(2-chlorobenzylamino)purine and an ethanolic solution (3 ml) of [(DMSO)_2_H][RuCl_4_NO(DMSO-κ*O*)] (DMSO = dimethyl sulfoxide) in a molar ratio of 2:1. The reaction mixture was stirred at room temperature for 5 min. After this time, a violet solution formed which was left to stand at room temperature. Violet crystals, suitable for single-crystal X-ray analysis, were deposited after slow evaporation of the solvent over a period of two days.

### Refinement

All H atoms were located in difference maps and refined using a riding model, with C–H distances of 0.95 and 0.99 Å, N–H distances of 0.88 Å, and with *U*_iso_(H) values of 1.2*U*_eq_(C,*N*). The O–H atoms were refined freely, see Table 1 for distances. The highest unassigned difference Fourier peak of 1.25 e Å^-3^ is located 0.84 Å from the Ru1 atom.

### Crystal data

**Table:**

(C_12_H_11_ClN_5_)[RuCl_4_(NO)(C_2_H_6_OS)]·H_2_O	*F*_000_ = 2512
*M**_r_* = 629.73	*D*_x_ = 1.844 Mg m^−^^3^
Orthorhombic, *P**b**c**a*	Mo *K*α radiation λ = 0.71073 Å
Hall symbol: -P 2ac 2ab	Cell parameters from 29289 reflections
*a* = 15.6229 (5) Å	θ = 2.6–31.9º
*b* = 12.8014 (4) Å	µ = 1.40 mm^−^^1^
*c* = 22.6866 (16) Å	*T* = 120 (2) K
*V* = 4537.2 (4) Å^3^	Prism, violet
*Z* = 8	0.40 × 0.30 × 0.25 mm

### Data collection

**Table:**

Oxford Diffraction Xcalibur2 diffractometer with CCD detector	3984 independent reflections
Radiation source: Enhance (Mo) X-ray Source	3588 reflections with *I* > 2σ(*I*)
Monochromator: graphite	*R*_int_ = 0.018
Detector resolution: 8.3611 pixels mm^-1^	θ_max_ = 25.0º
*T* = 120(2) K	θ_min_ = 2.6º
rotation method ω scans	*h* = −18→15
Absorption correction: multi-scan(CrysAlis RED; Oxford Diffraction, 2007)	*k* = −13→15
*T*_min_ = 0.604, *T*_max_ = 0.721	*l* = −26→26
36172 measured reflections	

### Refinement

**Table:**

Refinement on *F*^2^	Secondary atom site location: difference Fourier map
Least-squares matrix: full	Hydrogen site location: inferred from neighbouring sites
*R*[*F*^2^ > 2σ(*F*^2^)] = 0.031	H atoms treated by a mixture of independent and constrained refinement
*wR*(*F*^2^) = 0.079	*w* = 1/[σ^2^(*F*_o_^2^) + (0.0366*P*)^2^ + 10.7079*P*] where *P* = (*F*_o_^2^ + 2*F*_c_^2^)/3
*S* = 1.09	(Δ/σ)_max_ < 0.001
3984 reflections	Δρ_max_ = 1.25 e Å^−^^3^
279 parameters	Δρ_min_ = −0.58 e Å^−^^3^
2 restraints	Extinction correction: none
Primary atom site location: structure-invariant direct methods	

### Special details

**Table:**

Geometry. All e.s.d.'s (except the e.s.d. in the dihedral angle between two l.s. planes) are estimated using the full covariance matrix. The cell e.s.d.'s are taken into account individually in the estimation of e.s.d.'s in distances, angles and torsion angles; correlations between e.s.d.'s in cell parameters are only used when they are defined by crystal symmetry. An approximate (isotropic) treatment of cell e.s.d.'s is used for estimating e.s.d.'s involving l.s. planes.
Refinement. Refinement of *F*^2^ against ALL reflections. The weighted *R*-factor *wR* and goodness of fit *S* are based on *F*^2^, conventional *R*-factors *R* are based on *F*, with *F* set to zero for negative *F*^2^. The threshold expression of *F*^2^ > σ(*F*^2^) is used only for calculating *R*-factors(gt) *etc*. and is not relevant to the choice of reflections for refinement. *R*-factors based on *F*^2^ are statistically about twice as large as those based on *F*, and *R*- factors based on ALL data will be even larger.

### Fractional atomic coordinates and isotropic or equivalent isotropic displacement parameters (Å^2^)

**Table:**

	*x*	*y*	*z*	*U*_iso_*/*U*_eq_	
Ru1	0.616687 (16)	0.769508 (19)	0.208516 (10)	0.01845 (9)	
S1	0.47079 (7)	0.93802 (7)	0.19053 (4)	0.0406 (3)	
N1	0.57623 (16)	0.3182 (2)	0.47999 (11)	0.0210 (6)	
O1	0.52917 (14)	0.85527 (17)	0.16242 (10)	0.0250 (5)	
Cl2	0.67103 (5)	0.72294 (6)	0.11452 (3)	0.02083 (17)	
C2	0.5321 (2)	0.2412 (2)	0.45576 (14)	0.0225 (7)	
H2A	0.5052	0.2546	0.4190	0.027*	
N2	0.68824 (19)	0.6944 (2)	0.24609 (12)	0.0285 (6)	
O2	0.7319 (2)	0.6425 (2)	0.27444 (12)	0.0498 (8)	
O3	0.87301 (19)	0.6670 (2)	0.18872 (13)	0.0445 (7)	
Cl3	0.70383 (5)	0.92198 (7)	0.20765 (4)	0.0304 (2)	
N3	0.52234 (16)	0.1460 (2)	0.47885 (11)	0.0199 (5)	
H3A	0.4903	0.0986	0.4615	0.024*	
Cl4	0.55053 (6)	0.81931 (7)	0.29846 (3)	0.0326 (2)	
C4	0.56383 (19)	0.1251 (2)	0.53017 (13)	0.0183 (6)	
Cl5	0.51797 (6)	0.63075 (7)	0.20103 (4)	0.0317 (2)	
C5	0.61254 (19)	0.2011 (2)	0.55736 (14)	0.0198 (6)	
Cl6	0.63017 (6)	0.69071 (6)	0.46836 (4)	0.0329 (2)	
C6	0.61565 (18)	0.3025 (2)	0.53287 (14)	0.0189 (6)	
N6	0.65345 (17)	0.3834 (2)	0.55878 (12)	0.0224 (6)	
H6A	0.6813	0.3724	0.5919	0.027*	
N7	0.64498 (18)	0.1527 (2)	0.60684 (12)	0.0237 (6)	
H7A	0.6788	0.1809	0.6335	0.028*	
C8	0.6151 (2)	0.0543 (3)	0.60655 (15)	0.0237 (7)	
H8A	0.6287	0.0044	0.6361	0.028*	
N9	0.56465 (17)	0.0337 (2)	0.56098 (12)	0.0219 (6)	
C9	0.6513 (2)	0.4892 (2)	0.53527 (14)	0.0239 (7)	
H9A	0.6635	0.5389	0.5676	0.029*	
H9B	0.5928	0.5040	0.5207	0.029*	
C10	0.7143 (2)	0.5083 (2)	0.48574 (14)	0.0210 (7)	
C11	0.7108 (2)	0.6004 (2)	0.45299 (14)	0.0247 (7)	
C12	0.7666 (2)	0.6214 (3)	0.40738 (15)	0.0306 (8)	
H12A	0.7636	0.6859	0.3868	0.037*	
C13	0.8268 (2)	0.5472 (3)	0.39215 (16)	0.0358 (9)	
H13A	0.8648	0.5597	0.3602	0.043*	
C14	0.8317 (2)	0.4551 (3)	0.42324 (17)	0.0357 (9)	
H14A	0.8732	0.4041	0.4126	0.043*	
C15	0.7766 (2)	0.4362 (3)	0.47003 (15)	0.0281 (7)	
H15A	0.7815	0.3729	0.4916	0.034*	
C16	0.3684 (3)	0.8778 (5)	0.1860 (3)	0.094 (2)	
H16A	0.3664	0.8177	0.2128	0.142*	
H16B	0.3583	0.8542	0.1455	0.142*	
H16C	0.3242	0.9282	0.1973	0.142*	
C17	0.4570 (3)	1.0279 (3)	0.13257 (18)	0.0391 (9)	
H17A	0.5108	1.0654	0.1257	0.059*	
H17B	0.4121	1.0780	0.1431	0.059*	
H17C	0.4405	0.9905	0.0967	0.059*	
H3W	0.860 (3)	0.606 (2)	0.2071 (18)	0.050*	
H3V	0.9236 (18)	0.687 (3)	0.2050 (18)	0.050*	

### Atomic displacement parameters (Å^2^)

**Table:**

	*U*^11^	*U*^22^	*U*^33^	*U*^12^	*U*^13^	*U*^23^
Ru1	0.02301 (15)	0.01753 (15)	0.01482 (14)	−0.00037 (10)	−0.00315 (10)	−0.00055 (9)
S1	0.0678 (7)	0.0305 (5)	0.0233 (4)	0.0244 (5)	0.0070 (4)	−0.0014 (4)
N1	0.0198 (14)	0.0203 (14)	0.0229 (14)	−0.0022 (11)	−0.0009 (11)	0.0034 (11)
O1	0.0259 (12)	0.0285 (12)	0.0207 (11)	0.0062 (10)	−0.0028 (9)	−0.0053 (10)
Cl2	0.0188 (4)	0.0246 (4)	0.0191 (4)	0.0011 (3)	−0.0003 (3)	−0.0024 (3)
C2	0.0215 (16)	0.0227 (16)	0.0232 (17)	−0.0017 (13)	−0.0035 (13)	0.0038 (13)
N2	0.0396 (17)	0.0275 (15)	0.0183 (13)	0.0064 (14)	−0.0060 (13)	−0.0014 (12)
O2	0.062 (2)	0.0545 (18)	0.0328 (15)	0.0269 (16)	−0.0130 (14)	0.0043 (13)
O3	0.0407 (16)	0.0503 (18)	0.0424 (17)	−0.0016 (14)	−0.0104 (13)	0.0060 (14)
Cl3	0.0320 (5)	0.0241 (4)	0.0350 (5)	−0.0075 (3)	−0.0039 (4)	−0.0059 (3)
N3	0.0186 (13)	0.0181 (13)	0.0229 (14)	−0.0032 (11)	−0.0041 (11)	−0.0002 (11)
Cl4	0.0444 (5)	0.0349 (5)	0.0186 (4)	0.0014 (4)	0.0033 (4)	−0.0049 (3)
C4	0.0161 (15)	0.0169 (15)	0.0218 (16)	−0.0008 (12)	0.0021 (12)	−0.0004 (12)
Cl5	0.0378 (5)	0.0261 (4)	0.0312 (5)	−0.0117 (4)	0.0078 (4)	−0.0043 (3)
C5	0.0185 (15)	0.0197 (15)	0.0212 (15)	−0.0019 (13)	−0.0024 (12)	0.0025 (13)
Cl6	0.0488 (5)	0.0182 (4)	0.0316 (4)	0.0042 (4)	0.0020 (4)	−0.0021 (3)
C6	0.0148 (15)	0.0189 (15)	0.0231 (16)	−0.0004 (12)	0.0029 (12)	0.0012 (13)
N6	0.0259 (14)	0.0175 (13)	0.0238 (14)	−0.0049 (11)	−0.0042 (11)	0.0023 (11)
N7	0.0257 (14)	0.0228 (14)	0.0225 (14)	−0.0066 (12)	−0.0072 (11)	0.0039 (11)
C8	0.0257 (17)	0.0198 (16)	0.0257 (17)	−0.0049 (13)	−0.0057 (14)	0.0059 (13)
N9	0.0230 (14)	0.0187 (13)	0.0240 (14)	−0.0026 (11)	−0.0022 (11)	0.0041 (11)
C9	0.0279 (17)	0.0184 (16)	0.0255 (17)	−0.0014 (13)	0.0023 (14)	−0.0007 (13)
C10	0.0222 (16)	0.0188 (15)	0.0220 (16)	−0.0056 (13)	−0.0043 (13)	−0.0021 (13)
C11	0.0289 (18)	0.0214 (16)	0.0238 (17)	−0.0051 (14)	−0.0048 (14)	−0.0046 (13)
C12	0.037 (2)	0.0308 (19)	0.0239 (17)	−0.0133 (16)	−0.0009 (15)	0.0044 (14)
C13	0.0263 (19)	0.053 (2)	0.0280 (19)	−0.0101 (17)	0.0050 (15)	0.0023 (17)
C14	0.0235 (18)	0.045 (2)	0.038 (2)	0.0023 (16)	0.0033 (16)	−0.0018 (18)
C15	0.0237 (17)	0.0291 (18)	0.0317 (18)	−0.0014 (14)	−0.0010 (14)	0.0026 (15)
C16	0.050 (3)	0.075 (4)	0.158 (6)	0.028 (3)	0.062 (4)	0.052 (4)
C17	0.045 (2)	0.031 (2)	0.041 (2)	0.0084 (17)	0.0006 (18)	0.0047 (17)

### Geometric parameters (Å, °)

**Table:**

Ru1—N2	1.703 (3)	N6—H6A	0.8800
Ru1—O1	2.042 (2)	N7—C8	1.344 (4)
Ru1—Cl5	2.3585 (9)	N7—H7A	0.8800
Ru1—Cl2	2.3713 (8)	C8—N9	1.326 (4)
Ru1—Cl4	2.3746 (8)	C8—H8A	0.9500
Ru1—Cl3	2.3798 (8)	C9—C10	1.514 (4)
S1—O1	1.536 (2)	C9—H9A	0.9900
S1—C17	1.761 (4)	C9—H9B	0.9900
S1—C16	1.778 (6)	C10—C15	1.388 (5)
N1—C2	1.323 (4)	C10—C11	1.394 (5)
N1—C6	1.363 (4)	C11—C12	1.379 (5)
C2—N3	1.335 (4)	C12—C13	1.382 (5)
C2—H2A	0.9500	C12—H12A	0.9500
N2—O2	1.149 (4)	C13—C14	1.376 (5)
O3—H3W	0.904 (19)	C13—H13A	0.9500
O3—H3V	0.909 (19)	C14—C15	1.388 (5)
N3—C4	1.359 (4)	C14—H14A	0.9500
N3—H3A	0.8800	C15—H15A	0.9500
C4—N9	1.363 (4)	C16—H16A	0.9800
C4—C5	1.380 (4)	C16—H16B	0.9800
C5—N7	1.379 (4)	C16—H16C	0.9800
C5—C6	1.412 (5)	C17—H17A	0.9800
Cl6—C11	1.745 (3)	C17—H17B	0.9800
C6—N6	1.329 (4)	C17—H17C	0.9800
N6—C9	1.457 (4)		
			
N2—Ru1—O1	178.16 (12)	C5—N7—H7A	126.6
N2—Ru1—Cl5	92.30 (10)	N9—C8—N7	113.4 (3)
O1—Ru1—Cl5	86.00 (7)	N9—C8—H8A	123.3
N2—Ru1—Cl2	94.19 (10)	N7—C8—H8A	123.3
O1—Ru1—Cl2	85.09 (6)	C8—N9—C4	103.6 (3)
Cl5—Ru1—Cl2	88.86 (3)	N6—C9—C10	114.0 (3)
N2—Ru1—Cl4	90.41 (10)	N6—C9—H9A	108.7
O1—Ru1—Cl4	90.24 (7)	C10—C9—H9A	108.7
Cl5—Ru1—Cl4	88.82 (3)	N6—C9—H9B	108.7
Cl2—Ru1—Cl4	174.92 (3)	C10—C9—H9B	108.7
N2—Ru1—Cl3	95.26 (10)	H9A—C9—H9B	107.6
O1—Ru1—Cl3	86.44 (7)	C15—C10—C11	116.9 (3)
Cl5—Ru1—Cl3	172.39 (3)	C15—C10—C9	122.6 (3)
Cl2—Ru1—Cl3	89.65 (3)	C11—C10—C9	120.5 (3)
Cl4—Ru1—Cl3	92.06 (3)	C12—C11—C10	122.7 (3)
O1—S1—C17	102.31 (16)	C12—C11—Cl6	118.4 (3)
O1—S1—C16	102.2 (2)	C10—C11—Cl6	118.8 (3)
C17—S1—C16	97.5 (3)	C11—C12—C13	118.9 (3)
C2—N1—C6	119.4 (3)	C11—C12—H12A	120.5
S1—O1—Ru1	123.75 (13)	C13—C12—H12A	120.5
N1—C2—N3	125.2 (3)	C14—C13—C12	120.0 (3)
N1—C2—H2A	117.4	C14—C13—H13A	120.0
N3—C2—H2A	117.4	C12—C13—H13A	120.0
O2—N2—Ru1	175.0 (3)	C13—C14—C15	120.4 (4)
H3W—O3—H3V	104 (4)	C13—C14—H14A	119.8
C2—N3—C4	117.4 (3)	C15—C14—H14A	119.8
C2—N3—H3A	121.3	C14—C15—C10	121.1 (3)
C4—N3—H3A	121.3	C14—C15—H15A	119.5
N3—C4—N9	127.7 (3)	C10—C15—H15A	119.5
N3—C4—C5	120.5 (3)	S1—C16—H16A	109.5
N9—C4—C5	111.8 (3)	S1—C16—H16B	109.5
N7—C5—C4	104.5 (3)	H16A—C16—H16B	109.5
N7—C5—C6	136.1 (3)	S1—C16—H16C	109.5
C4—C5—C6	119.4 (3)	H16A—C16—H16C	109.5
N6—C6—N1	118.4 (3)	H16B—C16—H16C	109.5
N6—C6—C5	123.9 (3)	S1—C17—H17A	109.5
N1—C6—C5	117.8 (3)	S1—C17—H17B	109.5
C6—N6—C9	123.5 (3)	H17A—C17—H17B	109.5
C6—N6—H6A	118.2	S1—C17—H17C	109.5
C9—N6—H6A	118.2	H17A—C17—H17C	109.5
C8—N7—C5	106.8 (3)	H17B—C17—H17C	109.5
C8—N7—H7A	126.6		

### Hydrogen-bond geometry (Å, °)

**Table:**

*D*—H···*A*	*D*—H	H···*A*	*D*···*A*	*D*—H···*A*
N3—H3A···N9^i^	0.88	1.97	2.821 (4)	164
N6—H6A···O3^ii^	0.88	2.41	3.046 (4)	130
N6—H6A···Cl2^ii^	0.88	2.66	3.312 (3)	132
N7—H7A···O3^ii^	0.88	2.45	2.976 (4)	119
N7—H7A···Cl2^ii^	0.88	2.68	3.290 (3)	127
N7—H7A···Cl3^ii^	0.88	2.82	3.424 (3)	128
O3—H3W···Cl3^iii^	0.904 (19)	2.56 (3)	3.386 (3)	152 (4)
O3—H3V···Cl4^iv^	0.909 (19)	2.61 (3)	3.402 (3)	146 (4)
O3—H3V···Cl5^iv^	0.909 (19)	2.69 (3)	3.406 (3)	136 (4)

Symmetry codes: (i) −*x*+1, −*y*, −*z*+1; (ii) −*x*+3/2, −*y*+1, *z*+1/2; (iii) −*x*+3/2, *y*−1/2, *z*; (iv) *x*+1/2, *y*, −*z*+1/2.
